# Atherosclerosis affects calcium signalling in endothelial cells from apolipoprotein E knockout mice before plaque formation

**DOI:** 10.1016/j.ceca.2014.02.012

**Published:** 2014-03

**Authors:** Clodagh Prendergast, John Quayle, Theodor Burdyga, Susan Wray

**Affiliations:** Department of Cellular & Molecular Physiology, Institute of Translational Medicine, University of Liverpool, Liverpool, United Kingdom

**Keywords:** ApoE^−/−^, apolipoprotein E knockout, AUC, area under the curve, CCh, carbachol, MCD, methyl-β-cyclodextrin, ROS, reactive oxygen species, SOCE, store-operated Ca^2+^ entry, WHHL rabbits, Watanabe heritable hyperlipidaemic rabbits, WT, wildtype, Apolipoprotein-E knockout mice, Hypercholesterolaemia, Endothelium, Calcium signalling

## Abstract

Little is known about how hypercholesterolaemia affects Ca^2+^ signalling in the vasculature of ApoE^−/−^ mice, a model of atherosclerosis. Our objectives were therefore to determine (i) if hypercholesterolaemia alters Ca^2+^ signalling in aortic endothelial cells before overt atherosclerotic lesions occur, (ii) how Ca^2+^ signals are affected in older plaque-containing mice, and (iii) whether Ca^2+^ signalling changes were translated into contractility differences. Using confocal microscopy we found agonist-specific Ca^2+^ changes in endothelial cells. ATP responses were unchanged in ApoE^−/−^ cells and methyl-β-cyclodextrin, which lowers cholesterol, was without effect. In contrast, Ca^2+^ signals to carbachol were significantly increased in ApoE^−/−^ cells, an effect methyl-β-cyclodextrin reversed. Ca^2+^ signals were more oscillatory and store-operated Ca^2+^ entry decreased as mice aged and plaques formed. Despite clearly increased Ca^2+^ signals, aortic rings pre-contracted with phenylephrine had impaired relaxation to carbachol. This functional deficit increased with age, was not related to ROS generation, and could be partially rescued by methyl-β-cyclodextrin. In conclusion, carbachol-induced calcium signalling and handling are significantly altered in endothelial cells of ApoE^−/−^ mice before plaque development. We speculate that reduction in store-operated Ca^2+^ entry may result in less efficient activation of eNOS and thus explain the reduced relaxatory response to CCh, despite the enhanced Ca^2+^ response.

## 1 Introduction

Atherosclerosis is a leading cause of illness and death in the Western world. While a large literature exists describing many aspects of the atherosclerotic disease process ([1–5] for examples), there is much still to be clarified about the aetiology of plaque formation and of how elevated cholesterol levels can lead to this pathology. Apolipoprotein E knockout (ApoE^−/−^) mice exhibit chronic hypercholesterolaemia and go on to develop early and spontaneous atherosclerotic lesions [6–8] and as such, are a widely used model of atherosclerosis. In normal mice, the profile of cholesterol-containing plasma lipoproteins consists of predominantly high density lipoprotein (HDL) with only trace amounts of lower-density lipoproteins [6,9]. In contrast, ApoE^−/−^ mice have the reverse distribution: 80% of serum cholesterol is sequestered in lower density lipoproteins, while HDL-cholesterol is half the normal level. In addition, total plasma cholesterol levels are up to five times that of normal mice [6]. Thus, both the quantity of cholesterol and its distribution among lipoprotein fractions are shifted in the knockout mice to a pattern known to be associated in humans with risk of atherosclerosis.

An endothelial dysfunction, characterized by reduced ACh-mediated relaxation, has been reported in plaque-laden regions of the aorta [10]. In young mice, prior to plaque development, ACh-mediated relaxation appears unaltered, however, the basal availability of NO appears to be compromised before the onset of disease [11,12].

Intracellular Ca^2+^ signalling is vital in terms of both smooth muscle cell contraction and endothelial cell signalling and production of NO, yet very little is known about the Ca^2+^ signalling of the ApoE^−/−^ vascular endothelium, and how it is affected by this hypercholesterolaemic environment. In particular there is a lack of confocal microscopy studies, which have the benefit of allowing us to discriminate clearly between the Ca^2+^ signals emanating from endothelial cells and smooth muscle cells, while allowing the integrity of the endothelium:vascular smooth muscle interface to remain intact. Therefore, our aim was to determine whether chronic hypercholesterolaemia alters intracellular Ca^2+^ signalling and handling in aortic endothelial cells from young and old ApoE^−/−^ knockout mice using confocal microscopy. The use of animals that are 8–12 weeks old, before the knockouts show overt signs of plaque development, allowed us to look for the earliest signalling changes that may be occurring as a result of the dyslipidaemia and to compare with cells where plaques are well established. We also determined whether the Ca^2+^ signalling changes observed were translated into differences in contractility in functional aortic ring assays.

## 2 Materials and methods

### 2.1 Mice

Apolipoprotein E knockout mice (homozygotes) were obtained from Charles River and a breeding colony established in-house. Control C57BL/6J mice were obtained from Charles River as required. All were maintained on a normal chow diet. Young (8–12 weeks) or old (28–44 weeks) male mice were used in experiments.

### 2.2 Ethical approval

Mice were anaesthetized (CO_2_) and humanely killed by cervical dislocation in accordance with Schedule 1 of the UK Animals (Scientific Procedures) Act of 1986.

### 2.3 Plaque assessment

The timescale for the development of plaques in the ApoE^−/−^ aorta has been previously characterized by Nakashima et al. [8], indicating that monocyte adhesions are first observed from 8 weeks, foam cell lesions from 10 weeks and fibrous plaques after 20 weeks of age. The presence or absence of plaques in the aorta of young and old WT and ApoE^−/−^ mice was confirmed here using the lipophilic dye Sudan IV [13], which stains lipid deposits red. The aortic arch (down to 3 mm below the branching point of the subclavian artery) from 3 young WT and 4 young ApoE^−/−^ mice and 3 old WT and 4 old ApoE^−/−^ was removed and fixed in 10% neutral buffered formalin for 24 h. After this, tissues were rinsed 2× in PBS and stored in PBS at 4 °C until the staining procedure was carried out as follows: Each aorta was washed in 70% EtOH for 5 min, transferred to the Sudan IV solution (composition (1 L): 5 g Sudan IV, 500 ml acetone, 500 ml 70% EtOH) and gently agitated for 15 min and then destained in 80% EtOH for a further 5 min. Aortae were rinsed in PBS, dissected open along the outer edge of the arch and pinned out in a dissection dish containing PBS. Images were obtained using a dissecting microscope mounted with a Leica camera. These are confirmatory experiments and the data are not shown.

### 2.4 Confocal microscopy

The heart, with thoracic aorta attached, was removed and placed into a physiological salt solution of composition (mM): NaCl 154, KCl 5.6, MgSO_4_·7H_2_O 1.2, HEPES 10.9, glucose 8, and CaCl_2_ 2 (adjusted to pH 7.4). The aorta was cleared of adhering tissue, incubated with 23 μM Fluo-4 AM for 2 h at room temperature in the presence of 0.25% of the non-ionic detergent Pluronic F-127 and subsequently cut into strips (∼1 mm × 6 mm). The tissue was then placed in physiological salt solution to allow de-esterification of the dye. Strips were mounted, endothelium face down, under a small amount of isometric tension between two fixed aluminium foil clips at the bottom of the chamber, on the stage of an Olympus inverted microscope. The chamber was perfused with physiological salt solution at a constant flow rate (1 ml/min) and maintained at 30 °C. Experiments were performed using an Ultraview LCI spinning (Nipkow) disc, widefield confocal microscope (Perkin Elmer, Cambridge, UK), equipped with an Orca ER cooled CCD camera (Hamamatsu Photonics, UK) and either a 20× objective (N.A. 0.7) or a 60× water immersion objective (N.A. 1.20). Mean fluorescence intensity was measured on-line from regions of interest drawn over individual cells using UltraView software. Healthy-looking, well-loaded cells were chosen for analysis, with up to 16 individual cells analysed per tissue strip. Movement artefacts were rarely a problem when measuring from individual cells in a vessel under isometric tension. However, if substantial movement occurred, measurements were not made. The numerical data obtained were saved to an ASCII file for further analysis using Origin 7.0 software. The amplitude of the [Ca^2+^]_i_ signal was expressed as a normalized pseudo ratio of Fluo-4 fluorescence (*F*/*F*_0_). Once a stable baseline signal was established, the agonists carbachol (CCh) and ATP were applied and the resulting Ca^2+^ signals measured in terms of the amplitude of the initial peak response, the amplitude of the secondary plateau and area under the curve (measured over a 50 s range from the start of the Ca^2+^ rise). Data collected from plaque-laden vessels consist of measurements taken from individual cells adjacent to and some distance from plaques since the Ca^2+^ responses observed were similar regardless of the location of the cells examined (peak, plateau, AUC). Cyclopiazonic acid (CPA, 20 μM), a highly selective inhibitor of the Ca^2+^-ATPase of the intracellular Ca^2+^ stores [14,15], was used to measure the amount of releasable Ca^2+^ in the endoplasmic reticulum (ER) and gadolinium (Gd^3+^, 1 μM) was used to block store-operated Ca^2+^ entry [16].

### 2.5 Contractility

2 mm rings of thoracic aorta were mounted between 2 parallel stainless steel wires in 10 ml organ baths containing physiological salt solution (composition as above) maintained at 37 °C and continuously bubbled with oxygen. Rings were placed under an initial resting tension of 1.5 g and then allowed to stabilize for 30 min, during which time the organ bath fluid was replaced twice at 10 min intervals. Aortic rings were pre-contracted with 10 μM PE and once a stable contraction was achieved, cumulative concentration–response curves were obtained to the relaxatory agonist carbachol (CCh: 10 nM to 30 μM). In some experiments, single concentrations of agonist were applied instead (10 μM CCh, 20 μM SNAP). In additional contractility assays, 2 mm rings of aorta were mounted in a DMT dual wire myograph and maintained under the same conditions as described above. The rings were set to their normalized diameter according to the method of Mulvany and Halpern [17]. Briefly, the rings were stretched gradually until the calculated internal diameter reached a value equal to the internal circumference the vessel would have in vivo when fully relaxed and under a transmural pressure of 100 mmHg. Using this apparatus, the effect of superoxide dismutase (SOD, 100 and 300 U/ml) on the CCh response was assessed and the response to ATP examined.

### 2.6 Cholesterol manipulation

Membrane cholesterol was extracted using methyl-β-cyclodextrin (MCD), a cyclic oligosaccharide which sequesters cholesterol as previously described [18–20]. In confocal studies, 15 mM MCD dissolved in physiological solution (2% MCD) was applied to the tissue for 10 min, followed by a 20 min washout period, which has been shown to lower cholesterol by approximately 30% [20]. The same protocol was applied in organ bath studies.

### 2.7 Drugs and solutions

Unless otherwise specified, chemicals were obtained from Sigma (UK). Pluronic-F127 and Fluo-4 AM were obtained from Invitrogen. All compounds were made in aqueous solution apart from SNAP which was dissolved in DMSO (100 mM stock). In experiments using gadolinium (Gd^3+^), MgCl_2_ replaced MgSO_4_ in the physiological salt solution.

### 2.8 Statistics

Data are presented as mean ± SEM, where *n* = number of cells tested (confocal studies) or rings (organ bath studies), from a minimum of 3 aortae. The Student *t* test was used for statistical comparisons. A value of *p* < 0.05 was considered significant.

## 3 Results

No WT mice, young or old, exhibited atherosclerotic plaques in the aorta. This was confirmed by staining with Sudan IV (*n* = 3). In older ApoE^−/−^ mice, all aortae exhibited clear atherosclerotic plaques (all visible by eye and confirmed in 4 cases using Sudan IV staining). In young ApoE^−/−^ mice, no plaques were visible by eye, and Sudan IV staining revealed either no plaque or barely visible tiny areas presumably indicating where plaques would later form. These data confirm the pre-established timescale of plaque development [8] and data are not shown.

### 3.1 Endothelial cell [Ca^2+^]_i_ response

**Fig. 1:**
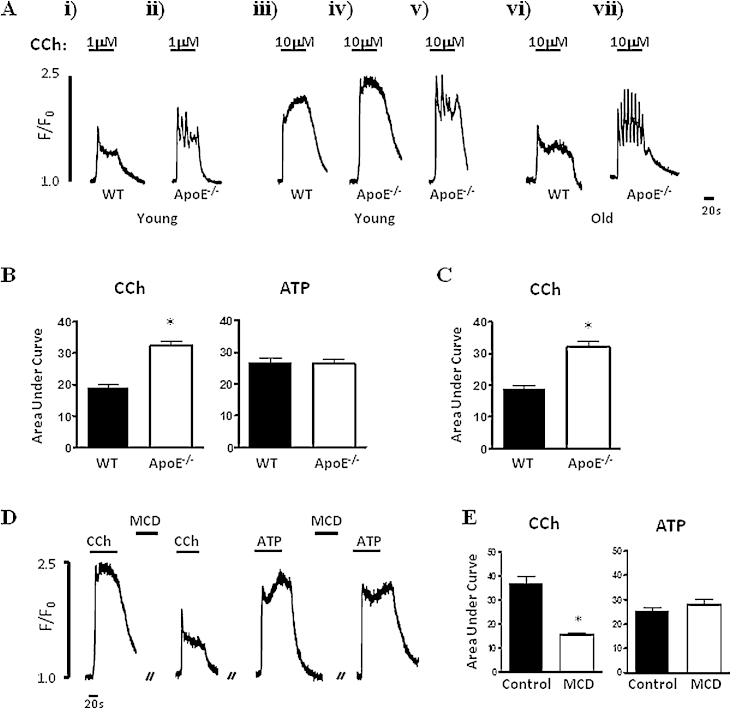
Intracellular Ca^2+^ responses to CCh and ATP in WT and ApoE^−/−^ aortic endothelial cells. (A) Experimental traces showing the Ca^2+^ response to 1 μM CCh (i and ii) and 10 μM CCh in endothelial cells from young (iii–v) and old (vi–vii) WT and ApoE^−/−^ mice. Panels vi and v demonstrate that Ca^2+^ responses to CCh could be sustained or oscillatory in nature. (B) Mean Ca^2+^ response to 10 μM CCh and 10 μM ATP in young WT and ApoE^−/−^ endothelial cells. (C) Mean Ca^2+^ response to 10 μM CCh in old WT and ApoE^−/−^ endothelial cells. (D) Experimental traces showing the response to 10 μM CCh is reduced after treatment with MCD in ApoE^−/−^ endothelial cells. The response to 10 μM ATP is unaltered. (E) Mean data showing effect of MCD on the CCh and ATP response in knockout endothelial cells from young mice. ^*^p < 0.05.

The effects of hypercholesterolaemia on intracellular Ca^2+^ signals to carbachol (CCh, 1 and 10 μM) and ATP (10 μM) were investigated in aortic endothelial cells. Both agonists produced a rapid upstroke of intracellular Ca^2+^ followed by a strongly sustained plateau response in WT and ApoE^−/−^ cells (Fig. 1A and D). In some ApoE^−/−^ preparations, CCh produced an oscillatory rather than sustained Ca^2+^ response; see Fig. 1Aiv and v for a comparison of the oscillatory and sustained responses to CCh in young ApoE^−/−^ endothelial cells. This will be described in more detail below. The Ca^2+^ responses were compared in terms of their initial peak response, the magnitude of the plateau phase and the area under the curve (AUC).

In young mice, the peak, plateau and AUC Ca^2+^ responses to CCh (10 μM) stimulation were all significantly larger in the ApoE^−/−^ endothelial cells (*n* = 116 cells/15 mice) compared to WT control responses (*n* = 128 cells/14 mice, Fig. 1Aiii–v and B). At a lower CCh concentration (1 μM), the peak (but not AUC) was also significantly increased in ApoE^−/−^ endothelial cells compared to WT (WT 0.60 ± 0.03, *n* = 104 cells/8 mice versus ApoE^−/−^ 0.76 ± 0.05, *n* = 96 cells/8 mice, Fig. 1Ai and ii). In the older ApoE^−/−^ mice, plaques were readily visible in all aortae examined. We found that the elevated Ca^2+^ response to CCh was still apparent and significant in these older tissues (WT *n* = 86 cells/6 mice, ApoE^−/−^ *n* = 118 cells/7 mice, Fig. 1Avi–vii and C).

In contrast, there were no changes in these parameters of the Ca^2+^ signals produced in response to ATP in ApoE^−/−^ endothelial cells (Fig. 1B, WT AUC 26.9 ± 1.3, *n* = 98 cells/9 mice versus ApoE^−/−^ AUC 26.3 ± 1.4, *n* = 91 cells/10 mice).

### 3.2 Modulation of cholesterol

If the differences in CCh signalling were directly related to the elevated amount of cholesterol present in the knockout mouse, then using methyl-β-cyclodextrin (MCD) to extract cholesterol from the cell membrane should reduce the CCh response in the ApoE^−/−^ aortic endothelial cells. When ApoE^−/−^ aortic strips were incubated with 2% MCD for 10 min, the endothelial cell Ca^2+^ response (peak, plateau and AUC responses) to CCh was significantly reduced (Fig. 1D and E, ApoE^−/−^ AUC 37.0 ± 2.7, *n* = 61 cells/6 mice versus MCD-treated 15.5 ± 0.9, *n* = 47 cells/4 mice). This was a selective effect, in that incubation with MCD did not alter the Ca^2+^ responses to CCh in the WT aortic endothelial cells (WT AUC 23.9 ± 1.4, *n* = 96 cells/9 mice versus MCD-treated 23.6 ± 1.5, *n* = 58 cells/5 mice). MCD did not alter the response to ATP in either WT or ApoE^−/−^ aortic endothelial cells (Fig. 1D and E).

### 3.3 Ca^2+^ handling

To gain mechanistic insight into how CCh-mediated Ca^2+^ responses are affected by hypercholesterolaemia, we evaluated (i) basal Ca^2+^ levels, (ii) the effect of SERCA inhibition on Ca^2+^ signalling and (iii) the store-operated Ca^2+^ entry (SOCE) component of the Ca^2+^ signals.

**Fig. 2:**
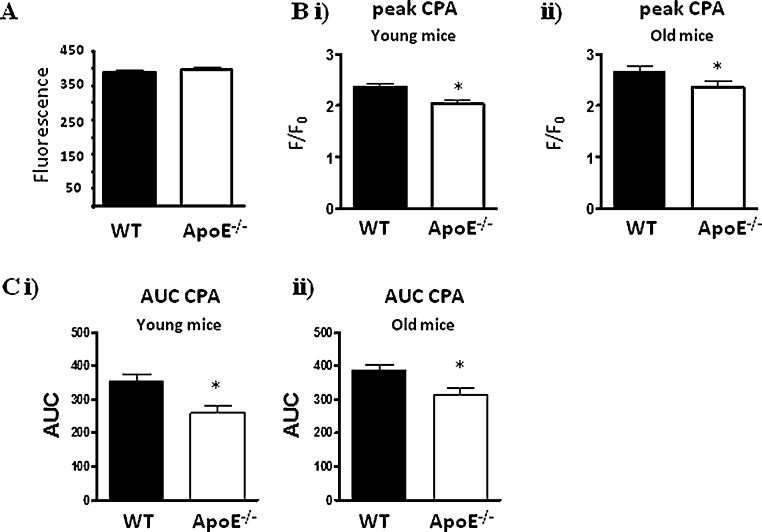
Measurement of basal endothelial cell Ca^2+^ and effect of CPA. (A) Mean basal fluorescence in young WT and ApoE^−/−^ endothelial cells. (B) Mean endothelial cell peak Ca^2+^ response to 20 μM CPA in (i) young WT and ApoE^−/−^ mice and (ii) old WT and ApoE^−/−^ mice. (C) Mean endothelial cell AUC Ca^2+^ response to 20 μM CPA in (i) young WT and ApoE^−/−^ mice and (ii) old WT and ApoE^−/−^ mice. ^*^p < 0.05.

To determine basal Ca^2+^ levels and compare them between ApoE^−/−^ and WT cells, we measured the basal fluorescence in a large number of WT and ApoE^−/−^ endothelial cells under identical experimental and recording conditions. No significant differences were observed (fluorescence (arbitrary units): 387 ± 4, *n* = 192 cells/10 mice versus 397 ± 4, *n* = 235 cells/13 mice; Fig. 2A).

The effect of SERCA inhibition on Ca^2+^ signalling was determined by administering CPA (20 μM). CPA produced a significantly larger rise in peak and AUC intracellular Ca^2+^ signals in the young WT mice compared to the ApoE^−/−^ mice (Fig. 2Bi and Ci, and mean data in Table 1), and this was also apparent in the old mice (Fig. 2Bii and Cii, and Table 1). When this experiment was repeated in zero Ca^2+^ conditions, the difference between WT and ApoE^−/−^ cells was removed (WT 1.17 ± 0.02, *n* = 62 cells/5 mice versus ApoE^−/−^ 1.12 ± 0.02, *n* = 58 cells/6 mice), suggesting that it is SOCE, not ER content, that is altered in the knockout mice.

**Fig. 3:**
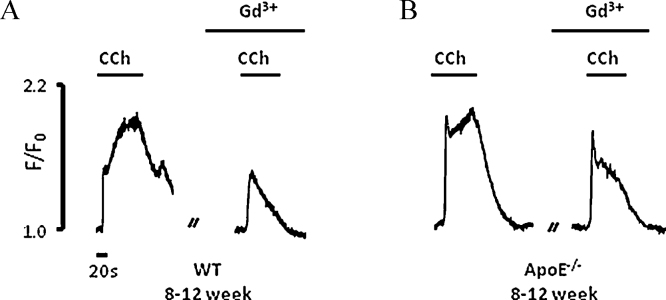
Effect of gadolinium on the CCh mediated Ca^2+^ response. Experimental traces showing the effect of Gd^3+^ treatment on the Ca^2+^ response to 10 μM CCh in (A) WT and (B) ApoE^−/−^ endothelial cells from young mice.

To assess SOCE we used Gd^3+^ (1 μM), a blocker of this form of Ca^2+^ entry and using a paired experiment protocol, compared Ca^2+^ responses to CCh in its absence and presence. The mean data are given in Table 1. In WT cells from young animals (Fig. 3A), the initial Ca^2+^ peak response was unaltered by Gd^3+^, however the size of the sustained plateau and area under the curve were greatly reduced. In ApoE^−/−^ cells from young animals, Gd^3+^ produced a small but significant reduction of the initial Ca^2+^ peak (Fig. 3B), and a large reduction of the Ca^2+^ plateau and AUC. The reduction in the size of the plateau phase with Gd^3+^ was however significantly greater in the WT cells than the ApoE^−/−^ cells (WT 67 ± 6% versus ApoE^−/−^ 45 ± 7%, *p* = 0.02). AUC was reduced to a similar degree in both WT and ApoE^−/−^ cells.

### 3.4 Sustained versus oscillatory Ca^2+^ signal

As mentioned above, the typical Ca^2+^ response to CCh in 8–12 week old endothelial cells was characterized by an initial peak and a sustained secondary plateau. In most cases, this characteristic response was observed in both WT and ApoE^−/−^ cells. However responses to CCh were more oscillatory in several preparations from young ApoE^−/−^ mice (Fig. 1Aii and v). While the increased percentage of endothelial cells producing an oscillating signal did not reach significance in young mice (WT: 1.8 ± 1.0%, *n* = 422 cells/11 mice versus ApoE^−/−^: 7.5 ± 4.2%, *n* = 351 cells/9 mice), in older mice, the difference was larger and significant (WT: 1.2 ± 0.8%, *n* = 355 cells/6 mice versus ApoE^−/−^: 9.8 ± 3.9%, *n* = 646 cells/5 mice, *p* = 0.04). After treatment with Gd^3+^, the number of old ApoE^−/−^ endothelial cells that generated an oscillatory signal increased significantly (from 8.1 ± 4.7% to 14.5 ± 5.7%, *n* = 3 mice, *p* = 0.034), while no difference was observed in WT cells after Gd^3+^ treatment. These observations, together with the CPA and Gd^3+^ data, further suggest that Ca^2+^ handling is altered in the knockout animal.

### 3.5 Measurement of functional responses in aortic rings

Since we had observed clear Ca^2+^ signalling differences in endothelial cells in the hypercholesterolaemic model, we were interested to determine whether these differences would be translated into functional differences in an aortic ring organ bath assay.

### 3.6 Relaxatory response

Rings of thoracic aorta were pre-contracted with phenylephrine (PE, 10 μM) and the relaxatory effects of carbachol determined. In young mice, the size of the PE response was not significantly different in WT and ApoE^−/−^ rings (*n* = 12–21 rings, data normalized to the KCl response in same tissue). However as the mice aged, the PE response became significantly smaller in the knockouts (20 weeks: WT 0.85 ± 0.04% versus ApoE^−/−^ 0.63 ± 0.07%, *n* = 14–16, *p* = 0.008 and 30 weeks: WT 0.76 ± 0.08% versus ApoE^−/−^ 0.53 ± 0.04%, *n* = 8–12, *p* = 0.009).

**Fig. 4:**
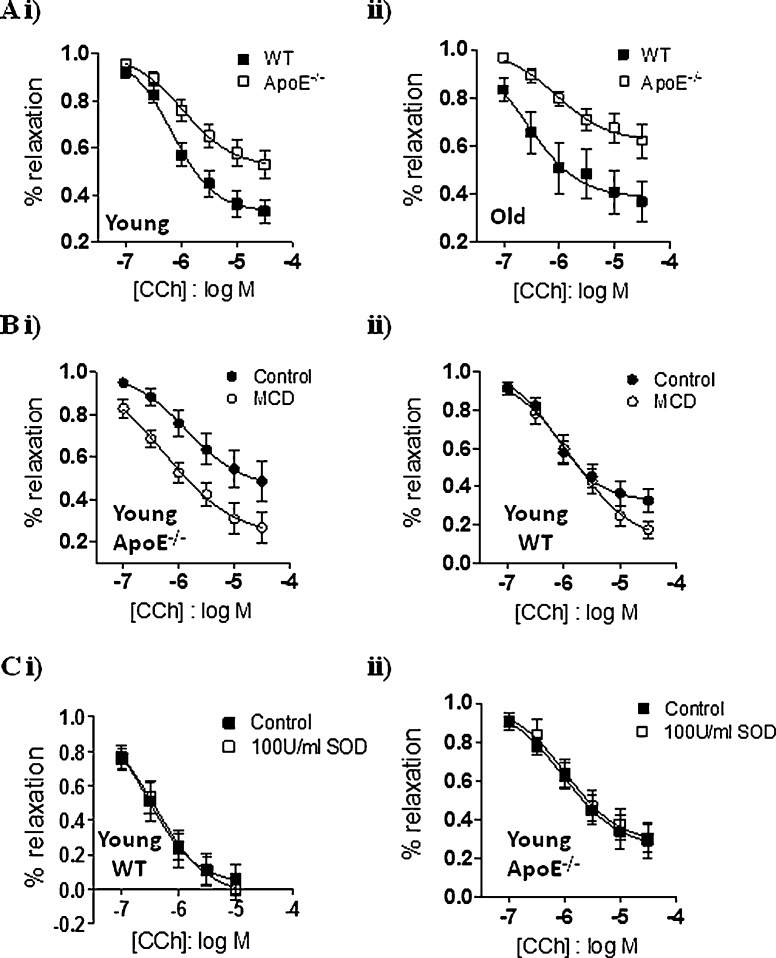
Dysfunctional vasorelaxation in knockout mice. (A) Concentration–response curves to CCh obtained in WT and ApoE^−/−^ aortic rings from (i) young and (ii) old mice. (B) Effect of MCD on the concentration–response curves to CCh obtained in (i) ApoE^−/−^ aortic rings and (ii) WT aortic rings, from young mice. (C) Concentration–response curves to CCh obtained in (i) WT and (ii) ApoE^−/−^ aortic rings in the absence and presence of 100 U/ml SOD.

Even in young ApoE^−/−^ animals, there was a clear deficit in the CCh-mediated relaxation. While the pEC_50_ remained unaltered (WT 6.16 ± 0.1 versus ApoE^−/−^ 5.97 ± 0.2), a significantly smaller relaxation was observed to 1, 3, 10, and 30 μM CCh in ApoE^−/−^ rings compared to WT (Fig. 4Ai, *n* = 12–14 rings from 5 to 7 mice). The response to CCh deteriorated further as the animals aged, with all concentrations of CCh (0.1–30 μM) now producing an attenuated relaxation in ApoE^−/−^ mice compared to WT (Fig. 4Aii, *n* = 9–11 rings from 3 to 5 mice), with little change in the pEC_50_ (WT 6.58 ± 0.23 versus ApoE^−/−^ 6.05 ± 0.24). Treatment with MCD to counteract the effects of the hypercholesterolaemia, led to a partial rescue of the CCh response (*n* = 7–9 rings from 3 to 5 mice, Fig. 4Bi), whereas MCD had no effect on the CCh-induced relaxant response in WT mice (*n* = 9–12 rings from 4 mice, Fig. 4Bii).

It has been suggested that hypercholesterolaemia results in the formation of reactive oxygen species (ROS), which may further result in reduced NO bioavailability [21]. In order to investigate whether elevated ROS activity is the cause of the reduced relaxatory response to CCh in ApoE^−/−^ aortic rings, we repeated the CCh curves in the presence of superoxide dismutase (SOD). Neither 100 nor 300 U/ml SOD altered the CCh dose-response curves in WT or ApoE^−/−^ rings (Fig. 4C, *n* = 6 rings/3 mice).

We also compared the ability of pre-contracted WT and ApoE^−/−^ rings to relax upon administration of SNAP, an NO donor. WT and ApoE^−/−^ rings (8–12 weeks) responded with a rapid and complete relaxation to 20 μM SNAP (WT 97 ± 2%, *n* = 19 rings/6 mice versus ApoE^−/−^ 97 ± 2%, *n* = 30 rings/10 mice). In older animals (20–25 weeks), the same ability to relax rapidly and completely to SNAP was observed (WT 95 ± 1%, *n* = 20 rings/7 mice versus ApoE^−/−^ 92 ± 2%, *n* = 22 rings/8 mice).

**Fig. 5:**
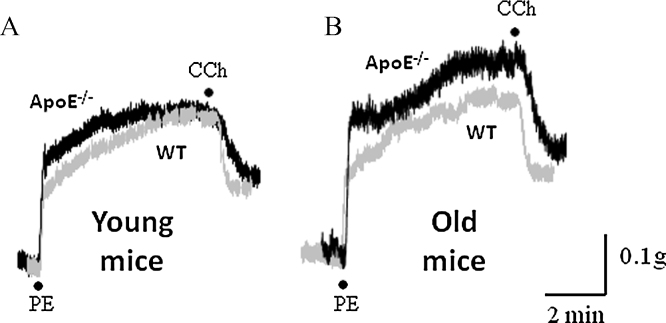
CCh half-time of relaxation altered in knockout mice. (A) Experimental traces showing the increased half-time of relaxation of CCh (10 μM) in ApoE^−/−^ aortic rings from young animals. (B) Experimental traces showing no significant difference in half-time of relaxation in old animals. Black traces represent ApoE^−/−^ and grey traces represent WT data.

We have already seen that there is a reduced maximal relaxation to CCh in ApoE^−/−^ rings. When we compared the half-time of relaxation of the CCh responses (in young mice), it was clear that this parameter was significantly slower in the knockouts compared to WT (WT 13.9 ± 0.9 s, *n* = 13 rings/5 mice versus ApoE^−/−^ 22.3 ± 1.8 s, *n* = 16 rings/8 mice, Fig. 5). The half-time of relaxation for SNAP was similar in both WT and ApoE^−/−^ rings (WT 13.2 ± 1.1 s, *n* = 19 rings/7 mice versus ApoE^−/−^ 14.7 ± 0.9 s, *n* = 18 rings/6 mice). In old animals (20–25 weeks), the half-time of relaxation for both CCh (WT 16.0 ± 1.1 s, *n* = 19 rings/7 mice versus ApoE^−/−^ 20.2 ± 2.3 s, *n* = 22 rings/8 mice) and SNAP (WT 12.7 ± 0.6 s, *n* = 14 rings/5 mice versus ApoE^−/−^ 14.7 ± 0.7 s, *n* = 18 rings/6 mice) were not significantly different in WT and ApoE^−/−^ mice.

When pre-contracted rings from WT mice were exposed to ATP (0.3 μM to 1 mM), no relaxation was observed, instead a small contractile response was obtained on top of the pre-contraction (*n* = 6 rings/3 mice). Rings from ApoE^−/−^ mice behaved identically (*n* = 6 rings/4 mice, data not shown). Relaxations to CCh were observed in all tissues that failed to relax to ATP stimulation, indicating that the endothelium was indeed intact and functional.

## 4 Discussion

Confocal microscopy confers several advantages compared to other techniques to measure Ca^2+^ in endothelial cells. We are able to distinguish clearly the Ca^2+^ signals emanating from endothelial cells, rather than providing a measure of a global Ca^2+^ signal from all cells including myocytes, and we are able to do so in intact vessel strips. By directly observing both the cells and their Ca^2+^ responses in the endothelium, measurements can be made with confidence that the endothelium has not been damaged. The current study is the first to use confocal microscopy to clearly isolate the endothelial cell responses and to observe specific and discrete Ca^2+^ signalling differences between ApoE^−/−^ and WT endothelial cells. Previous insightful studies measuring calcium in these mice used simultaneous measurement of force and global calcium concentration [22,23] and thus were unable to specifically measure endothelial cell responses. In our study of endothelial cells of ApoE^−/−^ mice, we found (i) no evidence for a rise of basal Ca^2+^, (ii) no evidence for altered ER releasable Ca^2+^, (iii) a reduction in SOCE, (iv) no alteration in the Ca^2+^ response to ATP but an exaggerated response to CCh and (v) evidence for an increasingly oscillatory Ca^2+^ response to CCh as disease progresses. These data suggest an adaptation to the hypercholesteremic environment is occurring in these cells. Consistent with previous reports of impaired vasorelaxation in atherosclerosis, we found impaired relaxation of the vessels to CCh, despite the increased Ca^2+^ signal. As discussed below, this may be a consequence of the reduced SOCE in the knockout endothelial cells. These findings give novel insight into the aetiology of altered vascular function occurring at the earliest stages of atherosclerotic disease.

We observed a significantly elevated intracellular Ca^2+^ response to CCh, but not ATP. This is the first reported example of an altered response to muscarinic receptor activation in young ApoE^−/−^ animals. This alteration, being present in young mice, indicates that it is a very early change that pre-dates the development of plaques and is related to the hypercholesterolaemic state, since modulation of cholesterol levels with MCD reversed the changes. Some previous studies in endothelial cells had shown ATP responses to be modified by experimental manipulation of cholesterol [23,24], but others reported that treatment with MCD failed to alter the ATP-mediated Ca^2+^ response [25] and this seems to be the case in our dyslipidaemic animal model. This elevated response to CCh could be due to an alteration in Ca^2+^ signalling and handling, or muscarinic receptor functional coupling and caveolar localization. These suggestions will be discussed further. Our finding of agonist-specific alterations in Ca^2+^ signalling suggests selective pathways are affected by dyslipidaemia, and that there is not a general downstream effect on all receptor-mediated responses. This agonist specificity has been observed previously after cholesterol manipulation [26–28].

Plasma membrane calcium ATPases (PMCA), Na^+^/Ca^2+^ exchangers (NCX) and SERCA pumps all function to remove Ca^2+^ from the cell [29]. If the activity of these transporters was down regulated by the chronically elevated cholesterol levels present in ApoE^−/−^ mice, then elevated intracellular Ca^2+^ levels would occur. However, we are confident this does not explain the elevated response to CCh seen here, as basal Ca^2+^ levels remain unaltered in ApoE^−/−^ mice and such changes would be expected to alter responses to both CCh and ATP, which we did not observe. Additionally, removal of cholesterol using MCD, did not affect the ATP response, all of which argues against the activity of these transporters being modified in the ApoE^−/−^ endothelial cells.

In addition to the specific increase in CCh-mediated Ca^2+^ signals in ApoE^−/−^ endothelial cells, CPA generated a reduced Ca^2+^ response in these cells, without a change in the amount of Ca^2+^ released from the internal store, suggesting a reduced role for SOCE in knockout cells. This was confirmed by the SOCE blocker Gd^3+^ having a significantly smaller inhibitory action on CCh-mediated responses in ApoE^−/−^ endothelial cells compared to WT. Interestingly, Van Assche et al. demonstrated that IP_3_-mediated SOCE was upregulated in aortic smooth muscle cells from these ApoE^−/−^ mice [22]. Together, these changes might be expected to blunt vasorelaxation and enhance vasoconstriction, leading to a significantly altered contractile state.

Caveolae are distinct lipid microdomains involved in the compartmentalization of many signalling molecules, including eNOS, and facilitating signal transduction. Caveolin-1 (cav-1) is abundantly expressed in the vasculature, binds cholesterol and is necessary for caveolae formation. Several components of Ca^2+^ regulation may be altered when caveolar function is modified. We and others have shown that cav-1 and cholesterol content of the plasma membrane play a role in Ca^2+^ regulation and SOCE [28,30–37]. Thus, caveolae and cav-1 play an important role in endothelial Ca^2+^ entry pathways. Such alterations could explain why SOCE is reduced and why responses to carbachol are increased in young ApoE^−/−^ aortic preparations, and suggests that the purinergic receptors may not couple to this pathway, as responses to ATP were not altered. There is existing evidence to support caveolae facilitating muscarinic receptor-induced Ca^2+^ mobilization. For example in airway smooth muscle, muscarinic M_3_R and Galpha(q/11) cofractionate with caveolin-1-rich membranes, and MCD in tracheal strips reduced the sensitivity to ACh [38]. There was no change in expression of muscarinic receptors, further strengthening the suggestion that it is coupling that is affected by caveolae number. The general importance of caveolae to cell signalling can be partially explained by the cav-1 scaffolding domain interacting with and regulating proteins which interact with and transduce G-coupled receptor effects, including, α-subunits of trimeric G proteins, PLC, the G protein RhoA and its downstream effector Rho kinase, and Ca^2+^-sensitive PKC isoforms. Thus caveolae can influence contraction and signalling not only by affecting Ca^2+^ regulation through co-localization of channel subunits and Ca^2+^ pumps, but also through modifying the receptor-evoked signalling pathways in endothelial cells and therefore fine tuning the responses to muscarinic agonists. This functional organization of caveolae provides for agonists that signal through common Ca^2+^ pathways, still giving rise to disparate responses.

Consistent with our CCh findings are data from Chen et al. [39], who examined rabbits on a high cholesterol diet, and found that ACh-mediated aortic endothelial Ca^2+^ signalling in areas adjacent to fatty streaks, became augmented in the second week of hypercholesterolaemia, but not in animals on a normal diet, and when the entire aorta was covered with fatty streaks in the fourth week of hypercholesterolaemia, endothelial calcium signalling was no longer elevated. Furthermore they also reported no changes in basal Ca^2+^ signals, as we found in the ApoE^−/−^ mice endothelial cells. Impaired muscarinic-mediated relaxation was also observed in the dyslipidaemic WHHL rabbit aorta [40,41]. The ATP response was not impaired, but interestingly showed some enhancement. We conclude that while basal Ca^2+^ mechanisms appear to function normally, it is carbachol-induced Ca^2+^ responses that consistently show alteration in the face of chronic hypercholesterolaemia. It should be noted that Kaiser et al. [23] and Paffett et al. [24] showed P2Y receptor responses to be altered by MCD in endothelial cells from guinea pig aorta and rat pulmonary artery, respectively. However, Beliveau and Guillemette [25] did not see this effect in bovine aortic endothelial cells, and we do not see an effect on the ATP response in this study, indicating that responses may be species specific.

Caveolae, and hence cholesterol levels, are intimately connected to NO signalling. Enhanced coupling of cav-1 with eNOS is associated with diminished production of NO in chronic hypoxia [42]. Endothelial Ca^2+^ signals lead to production of nitric oxide, which not only causes vasorelaxation but also has powerful anti-inflammatory and anti-thrombotic properties [43]. Thus we speculate that the enhanced endothelial calcium signals to CCh we found in the early stages of dyslipaedemia, may be a protective response against atherosclerosis. This may be analogous to the protective changes that occur in early stages of hypertension; where the endothelial nitric oxide pathway and the amount of acetylcholine-evoked NO release is elevated in spontaneously hypertensive rats [44]. Once intracellular calcium signalling reduces in the ApoE^−/−^ mice, the protective role of NO will fail. Our studies showed that in the aortae of ApoE^−/−^-deficient mice, endothelium-dependent relaxation becomes progressively retarded with age. We did not find however normal relaxation in young ApoE^−/−^ aortic preparations, despite the increased level of Ca^2+^ signalling. The relaxatory response was reduced and the half-time of relaxation for CCh was slower in knockout mice, whereas the relaxatory response to SNAP (and the half-time of relaxation) was unaltered. This may be because the compensation is inadequate in the face of other damaging changes. However our data also point to a role of altered Ca^2+^ signals.

Lin et al. [45] demonstrated that eNOS is better activated by Ca^2+^ that enters the cell via SOCE than Ca^2+^ released from the internal store. The reduced SOCE that we observed in both young and old knockout mice may therefore result in less efficient activation of eNOS and thus explain the reduced relaxatory response to CCh, despite an enhanced Ca^2+^ response. Our experiments with SNAP show that if NO is supplied by this route, then the vessels from the ApoE^−/−^ mice relaxed without impairment. This strongly suggests that impairment in NO generation is a key change provoking poor relaxation in hypercholesterolaemic environments. In studies of human vessels, decreased function is also reported prior to plaque formation [46,47]. Endothelial dysfunction in diseased arteries is often attributed to an increase in the production of superoxide anions, leading to a reduced bioavailability of NO [21]. However the inability of superoxide dismutase to alter the CCh-mediated relaxatory response in ApoE^−/−^ aortic rings suggests this is not the case in our study. Hypercholesterolaemia has also been shown to reduce the expression of gap junction connexins (Cx37, Cx40 and Cx43 [48,49]) and inhibition of gap junctional communication can result in dysfunction of endothelial-mediated vasodilation [50–52]. However, despite demonstrating that aortic endothelial cells in plaque-laden areas from 9 month old ApoE^−/−^ mice did indeed lack gap junctions, Yeh et al. [49] showed that adjacent plaque-free areas looked normal. Additionally, in 3 month old ApoE^−/−^ mice, the number of gap junctions was reduced but not significantly so. Therefore in ApoE^−/−^ mice hypercholesterolaemia alone does not appear sufficient to significantly affect gap junctions and therefore they are unlikely to be contributing to the reduced vasorelaxation we observe. An alternative explanation may involve the more oscillatory and less sustained nature of the Ca^2+^ signals in endothelial cells of ApoE^−/−^ mice compared to WT. This phenomenon is more apparent as the mice age. Oscillations were also increased when preparations were treated with gadolinium, further suggesting that as the mice age and SOCE declines, signalling will be more oscillatory. Potentially, this oscillatory pattern of Ca^2+^ signalling may be less able to stimulate NO formation and contribute to the deficient relaxatory response observed, though this, and the relation to decreased SOCE, remains speculative. There is support for the idea however in the study of Krupp et al. [53], where they show that the loss of a sustained Ca^2+^ signal underlies a deficit in NO production in pre-eclamptic umbilical vein endothelium.

Our data with ATP are challenging, as we found no ATP-mediated relaxatory effect in ApoE^−/−^ or WT aortic rings. While the literature on purinergic responses is varied, some authors using ATP in ApoE^−/−^ mice have reported relaxation [10]. Our lack of relaxation cannot be explained by endothelial damage as relaxations to CCh were observed in all tissues that failed to relax to ATP stimulation. It may be that the contractile response to purinergic stimulation overwhelms any relaxation in our preparations. We would also expect SOCE alterations with hypercholesterolaemia to have effects on the ATP signals, but this was not the case. We suggest that non-caveolae mediated Ca^2+^ entry may play a larger role in ATP signalling than it does in CCh responses. There is some support for this in our data with zero Ca^2+^ (not shown). Removal of extracellular Ca^2+^ reduced the peak Ca^2+^ response to ATP by about 34% in WT and 39% in ApoE^−/−^ mice. For CCh these respective figures were 28% in WT but 50% in ApoE^−/−^ mice. If this can be equated to the CCh response in ApoE^−/−^ mice being comprised more from Ca^2+^ entry, then the reduction in SOCE may have more impact on CCh than ATP.

In conclusion, this is the first study to measure Ca^2+^ responses in ApoE^−/−^ endothelial cells using confocal microscopy and we have identified specific and discrete Ca^2+^ signalling and handling changes, even before overt plaque development. As a result of the chronic hypercholesterolaemia, carbachol-mediated Ca^2+^ responses are enhanced, while ATP-mediated responses are unchanged. A reduction in SOCE has also been observed, which we speculate may lead to poorer activation of eNOS and thus explain the reduced vasorelaxation in ApoE^−/−^ tissues despite an elevated Ca^2+^ response. Thus our data add to those of others suggesting how cholesterol levels and caveolae may affect the regulation of specific receptor pathways and Ca^2+^ signalling processes. These changes, the earliest derangements of Ca^2+^ signalling in dyslipidaemia may be key to atherosclerotic progression.

## Figures and Tables

**Table 1:** Intracellular calcium responses after inhibition of SERCA and SOCE. Mean data demonstrating the size of the response to 20 μM CPA in young and old WT and ApoE^−/−^ endothelial cells (upper table) and the effect of 1 μM Gd^3+^ on the CCh-mediated Ca^2+^ response in WT and ApoE^−/−^ endothelial cells from young animals (lower table).

Animal	Age	Added to bath	Ca^2+^ response (*F*/*F*_0_)		*n* (cells)
			Peak	AUC	
WT	Young	CPA	2.36 ± 0.06	356 ± 19	65
ApoE^−/−^	Young	CPA	2.04 ± 0.07	260 ± 20	84
WT	Old	CPA	2.67 ± 0.09	386 ± 18	72
ApoE^−/−^	Old	CPA	2.36 ± 0.12	313 ± 19	57

Animal	Age	Added to bath	Ca^2+^ response (*F*/*F*_0_)			*n* (cells)
			Peak	Plateau	AUC	
WT	Young	CCh	0.59 ± 0.03	0.71 ± 0.06	22.8 ± 1.5	35
WT	Young	CCh and Gd^3+^	0.54 ± 0.03	0.19 ± 0.03	11.7 ± 0.8	35
ApoE^−/−^	Young	CCh	0.99 ± 0.07	0.86 ± 0.06	30.9 ± 2.0	33
ApoE^−/−^	Young	CCh and Gd^3+^	0.80 ± 0.05	0.44 ± 0.03	18.1 ± 1.0	33
